# Bellevue Hospital

**Published:** 1849-05

**Authors:** 


### BELLEVUE HOSPITAL.

The Annalist notices improvements in this institution which will add to its usefulness. A new operating theater and clinical lecture room has been erected, and was formally opened and dedicated on the 2d of March. An address by Dr. Reese, the Resident Physician, was delivered on the occasion. Clinical instruction will form a part of the duties of the visiting physicians. Our correspondent, Dr. B. F. Wendel, whose reports from this hospital will be remembered by our readers, has again been appointed to a place in the institution, and his reports will be resumed.

